# Decentralised same day test and treatment of hepatitis C levering existing peer support networks among men who inject drugs: feasibility and effectiveness

**DOI:** 10.1186/s12954-024-01001-1

**Published:** 2024-05-20

**Authors:** Nalinikanta Rajkumar, Lokeshwar Singh Khumukcham, Dhabali Thangjam, Surender Singh, Giten Khwairakpam, Sonjelle Shilton, Amit Goel

**Affiliations:** 1 Community Network for Empowerment (CoNE), Imphal, Manipur India; 2 Jawaharlal Nehru Institute of Medical Sciences, Imphal, Manipur India; 3 BABINA Diagnostics, Imphal, Manipur India; 4 Sanjay Gandhi Postgraduate Institute of Medical Sciences, Lucknow, India; 5 TREAT Asia/amfAR, Bangkok, Thailand; 6 FIND, The Global Diagnostic Alliance, Geneva, Switzerland

**Keywords:** Hepatitis C, Hepatitis C, People who inject drugs, Same day test and treat, HCV, HBV, Drug treatment

## Abstract

**Background:**

Prevalence of hepatitis C virus (HCV) infection among people who inject drugs in the state of Manipur, India, is 43%; however, access to care is poor. We piloted a Community-led and comprehensive hepatitis care model that included same-day HCV treatment at drug treatment centres.

**Methods:**

Screening was conducted through venipuncture samples collected by community peer PWID, using HCV antibody (HCV Ab) rapid screening and hepatitis B virus (HBV) surface antigen (HBsAg) rapid diagnostic tests. Reactive HCV Ab samples were tested for HCV RNA using near point-of-care Truenat® HCV on Truelab® Quattro. Eligible HCV RNA-positive participants were treated on the same day using direct-acting antivirals and followed for sustained virologic response (SVR). HBsAg-negative participants received rapid HBV vaccination regimen while those positive for HBsAg were tested for DNA and referred for treatment.

**Results:**

Between November 2021 and August 2022, 643 individuals were approached and 503 consented and were screened. All screened were males with history of injection drug use, and a median age of 27 years (IQR 23–32). Of the 241 (47.9%) HCV Ab reactive all underwent RNA testing and 156 (64.7%) were RNA detectable. Of those with viraemia, 155 (99.4%) were initiated on treatment with 153 (98.1%) on same day, with 2 (1.2%) HBsAg positive and waiting for HBV DNA results. Among those 153, median time from HCV Ab screening to treatment was 6 h 38 min (IQR 5 h 42 min–8 h 23 min). In total 155 (100%) completed HCV treatment, of those 148 (95.5%) completed SVR testing and 130 (87.8%) achieved SVR12. 27 (5%) participants were HBsAg-positive, 3 (11.1%) were also living with HCV viraemia; 443 (97.6%) were eligible for vaccination and 436 (98.4%) received all 3 vaccine doses.

**Conclusion:**

Community-led hepatitis care incorporating same day “test and treat” for HCV was feasible and effective. HBV screening identified a large proportion who were unvaccinated. Peer support extended resulted in ensuring compliance to care and treatment cascade and completing all the three doses of HBV vaccination. As the screening, diagnostics infrastructure and vaccine are available in most countries with national viral hepatitis programs also in place, our model can be adapted or replicated to progress towards global elimination targets.

## Background

Hepatitis B and C are major global health problems. Globally, an estimated 296 million people live with hepatitis B infection (HBV) and 58 million people live with hepatitis C infection (HCV) [1]. In their chronic forms, both HBV and HCV can cause liver disease and liver cancer, resulting in substantial morbidity and mortality. Rates of HBV and HCV are particularly high among certain populations, including people who inject drugs (PWID). Direct-acting antivirals (DAAs) for HCV have high cure rates while vaccination against HBV provides long-term protection against the infection. Unfortunately, most people with HBV and HCV are unaware that they are living with the virus as symptoms of the infection also do not appear until late-stage of liver disease.

A major issue in ensuring that people living with HBV and HCV are diagnosed and treated is that the care cascade for HBV and HCV is complex, requiring different diagnostic tests and linkage of patients for clinical assessments, treatments and vaccination. Diagnosis of HCV infection, with current standard of care, requires an initial rapid test for HCV antibodies (HCV Ab) to identify exposure to the virus, followed by a test for HCV RNA, to confirm viraemic infection. Diagnosis of HBV requires an initial rapid diagnostic test for HBV surface antigen (HBsAg), followed by an HBV DNA test to evaluate for viraemic infection [2].

In India, around 40 million people are estimated to have HBV [3], and an estimated 12–18 million people have HCV [4]. In 2018, India launched The National Viral Hepatitis Control Program (NVHCP), aiming to achieve elimination of HCV by 2030, and achieve significant reductions in morbidity and mortality associated with HCV and HBV, through enhanced community awareness, early diagnosis and management, developing standard diagnostic and treatment protocols, and developing linkages with existing national programmes [5].

As rates of HCV and HBV infection are particularly high among PWID, ensuring these populations have access to prevention tools, diagnostic testing, treatment and follow-up is a priority to meet India’s hepatitis elimination goals. In the state of Manipur, 43% of PWID have exposure to HCV [6] while earlier studies had observed HCV Ab prevalence of 98% [7]. However, access to and uptake of HCV care is poor, largely due to lengthy pre-treatment processes. Under the national programme, a person needs to visit a healthcare centre multiple times in between screening to ascertaining if a cure has been achieved. Studies have shown that less than 6% of PWID had seen a doctor for care and less than 3% had initiated treatment [8] which seem to have seen not much improvement from earlier studies indicating only 5.2% of PWID had accessed testing [9]. In Manipur, linkage to care is also low. Among 8388 PWID and PLHIV volunteering for HCV Ab screening, 47% (n = 3986) were reactive to HCV Ab, but only 51% (n = 2047) presented for HCV RNA testing. Of the individuals undergoing RNA testing, 78% (n = 1612) were confirmed to have viraemia. However, treatment was initiated among only 55% (n = 889) of the cases, with 45% (n = 723) of patients being lost in between the diagnosis processes and treatment initiation [10].

Globally, it is accepted that the diagnostic algorithm and strategies to screen, prevent and treat HCV and HBV have to be simplified, if we are to achieve progress towards global elimination targets [11]. To improve community-based care models for HBV and HCV, we piloted a Community-led, comprehensive, simplified hepatitis care model that includes same-day HCV testing and treatment initiation (“test and treat”) at drug treatment centres in Manipur, to expand access to care for chronic hepatitis.

## Methods

### Partners

#### Community network for empowerment (CoNE)

**Fig. 1:**
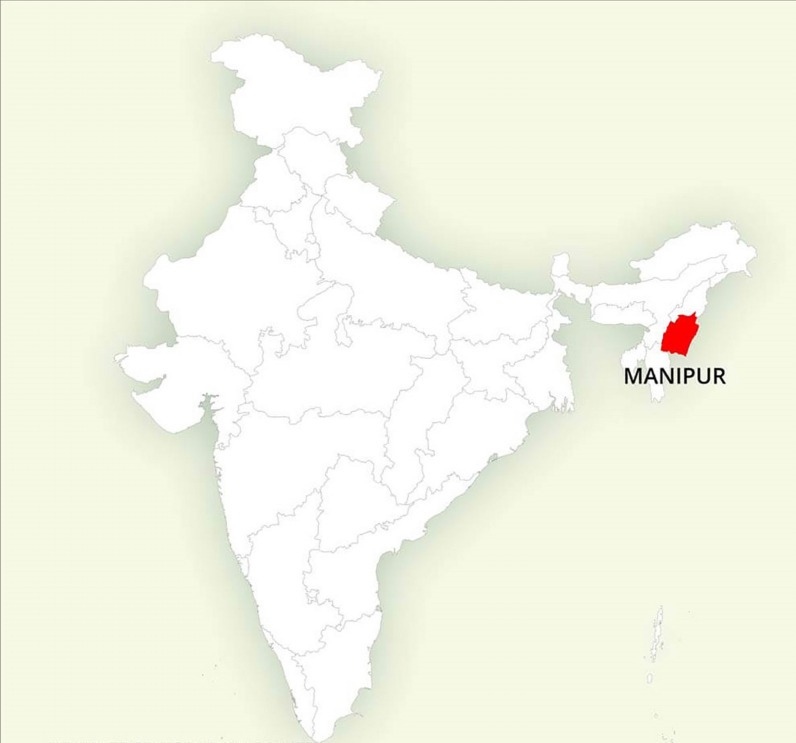
Location of Manipur in India

Is a non-profit organization of PWID registered in India. The organization supports HBV and HCV related preventive and curative services, for PLHIV and PWID, in the state of Manipur, India. It also engages with the state and national government to assist them in drafting and implementing policy and health programs for PWID and PLHIV. The organization has provided HCV screening, diagnosis, and treatment services since 2014. Being a PWID network, all the members of the organization have lived experience of drug use. The peer workers in this study came from the staff who are in the payroll of CoNE. They were trained on HBV and HCV by TREAT Asia/amfAR and Sanjay Gandhi Postgraduate Institute of Medical Sciences. The pre-test counselling, phlebotomy, post-test counselling, treatment education and adherence support was provided by the peer staff of CoNE, along with the DTC staff. For this study, CoNE had received technical support from TREAT Asia/amfAR, financial support from by FIND-the global alliance for diagnostics and collaboration from the Manipur state management unit of NVHCP, Model Treatment Centre, Jawaharlal Nehru Institute of Medical Sciences, and BABINA Diagnostics (Fig. 1).

### Study setting

**Fig. 2:**
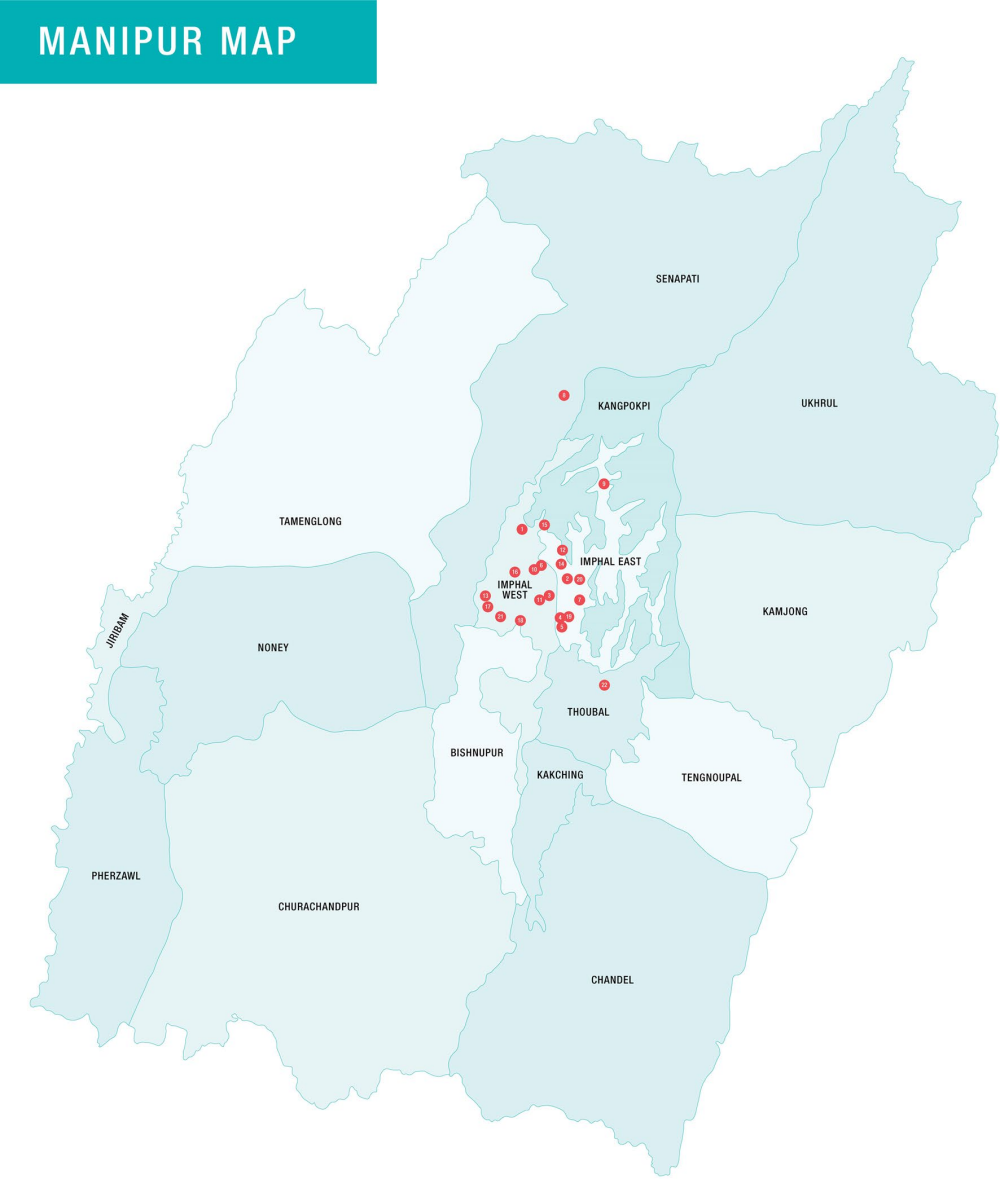
Location of the drug treatment centres

Manipur is a state in the north eastern region of India (Fig. 1), which has 16 districts and a population of ~ 2.8 million. Survey conducted by the Ministry of Social Justice and Empowerment in 2019, estimated approximately 34,000 PWID in the state of Manipur [12]. HCV prevalence among PWID in the state was reported at 74% [13] while recent studies have shown to be at 43% [6]. As a part of their service between November 2021 and August 2022, CoNE approached PWID registered in 22 Drug treatment centres (DTC) in Manipur, India from a total 91 approximately in the state. These DTC are centres where people who use drugs undergo treatment voluntarily for a finite period of time, usually 3 months. The first 7 days are detoxification period, where a person is prescribed with benzodiazepine or opioid medication to get over cravings of hard drugs. After the detoxification period, vitamin supplements or other medications for health recovery are prescribed by the physician. The centres were located in four districts (Imphal East, Imphal West, Thoubal and Senapati) of Manipur and were selected based on the willingness of the centres to participate, existing work relationship with CoNE, and convenience (Fig. 2). These DTC exclusively cater to male PWID, are mostly based on principles of abstinence, and engage a physician and nurse who visits once or twice a week for routine health check-ups of the clients undergoing treatment for drug use. There are only 3 DTC for women who use drugs in the state of Manipur with a total capacity of 45 beds, which is insufficient with reference to the need. These centers are run by women and have similar healthcare services and duration for treatment with that of the male ones.

### Aims and objectives

The primary objectives of the study were to:

- To show enhanced linkages from HCV diagnosis to treatment initiation among male PWID under the national HCV programme.
-  To develop an HCV “test and treat” model of care for male PWID that is replicable and amenable to scaled implementation under the national hepatitis control programme.

### Eligibility criteria

To be eligible for the study:

-  Participants of ≥ 18 years of age
-  Individuals who were residing at one of the 22 DTC
-  Individuals who attended the awareness sessions held at the 22 DTC
-  Willing to provide informed consent to participate

Exclusion criteria:

- Individuals who had previously undergone HCV DAA treatment

### Same day test and treat strategy

Treatment criteria for participants who were found to have HCV viremia followed the NVHCP guidelines. Participants who had detectable HCV RNA and also a positive HBsAg Rapid diagnostic test (RDT) result were required to undergo HBV DNA testing to determine if they were eligible for HCV DAA treatment based on the National Guidelines. Treatment eligibility was as follows:

To be eligible for HCV treatment:

- Participants with chronic HCV infection with any genotype, confirmed by a HCV Ab reactive test and detectable HCV RNA.
- Participants willing to start therapy and adhere to the study schedule.

Exclusion for HCV treatment:

- Individuals that have decompensated liver cirrhosis.
- A history of comorbidity or medication that in the opinion of the study physician, made the individual unsuitable for treatment administration
-  Individuals taking the antiarrhythmic medication amiodarone

### Study procedures

**Fig. 3:**
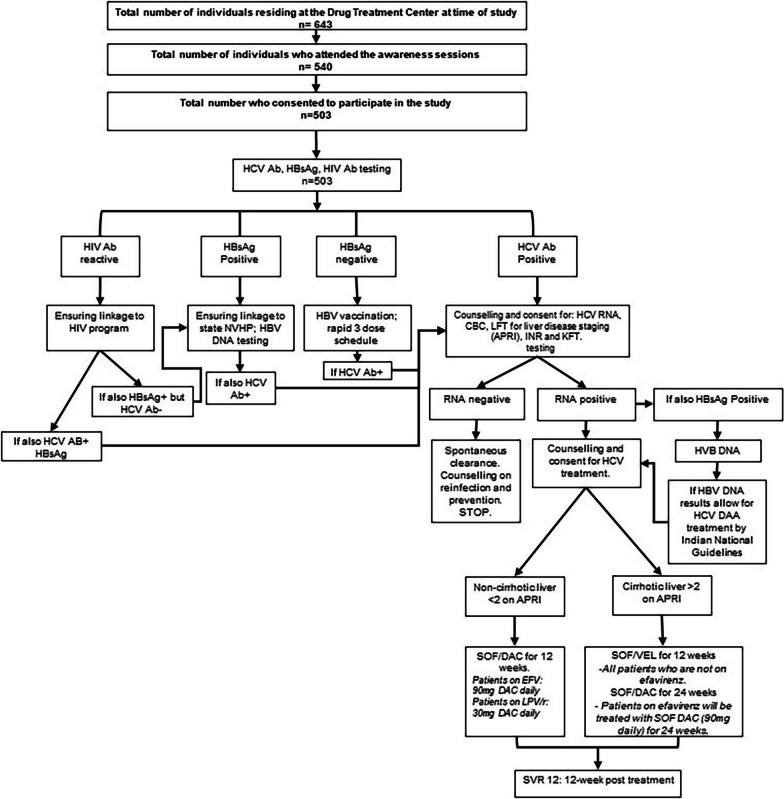
Screening, diagnosis and treatment flow chart Drug Treatment Centres

The entire process was completed in following steps as shown in Fig. 3:

- Conducting awareness sessions by the peer PWID at each DTC to educate the clients about various aspects of HBV and HCV with focus on risk factor, route of transmission, disease burden among PWID, diagnosis, treatment, and facilities available in NVHCP. They were also requested to participate in the “test and treat” program which was planned for the next day.
- Eligible participants were enrolled after written informed consent and 2.0 ml of blood was drawn by venipuncture; drops of whole blood were used for HCV Ab screening and HBsAg with rapid diagnostic test kit provided by NVHCP and their results were made available in 15–20 min. The HCV Ab screening test was performed with Standard Q HCV Ab Test® (Manufacturer SD Biosensor, Gurugram, India) and the HBsAg diagnostic test was performed with the Standard Q (SD Biosensor, Mfd. site Gurugram, India). Remaining sample was stored and transported to integrated Counselling and Testing Centre (ICTC) for HIV testing.
- A second blood specimen of 6.0 ml volume was collected on the same day from HCV Ab reactive participants through venepuncture by the peer PWID and was transferred to an offsite laboratory for HCV RNA quantitation, complete blood count, liver function test, serum creatinine, and prothrombin time INR. The HCV RNA quantitative assay was performed with Truenat® HCV on Truelab® Quattro (Molbio Diagnostics, Verna, India) with sample extractor Trueprep/Auto V2. The results of the HCV RNA and other pre-treatment test were made available on the same day. This platform is made in India, near point-of-care molecular testing, requires minimal infrastructure, minimally trained technicians and provides an end-to-end solution with results available in under an hour.
- Participants with confirmed chronic HCV infection and meeting treatment eligibility requirements were linked on the same day to the Model Treatment Center at Jawaharlal Nehru Institute of Medical Sciences under the NVHCP, and were offered treatment on the same day. AST-Platelet ratio index (APRI) was used to determine cirrhosis and those with APRI > 2.0 were presumed to have cirrhosis. Those with compensated cirrhosis were treated with sofosbuvir/velpatasvir (400 mg/100 mg) combination for 12 weeks whereas those without cirrhosis were treated with sofosbuvir (400 mg) plus daclatasvir (60 mg) for 12 weeks as per the national guidelines.
- The entire cascade of HCV Ab screening, RNA testing, complete laboratory evaluation, and start of antiviral treatment, was completed in the same day if the participant was eligible for same day treatment initiation. Participants were followed until test of cure, defined as sustained virologic response at 12 weeks post-treatment (SVR12).
- Participants who tested HBsAg positive were linked to the NVCHP for further assessments including HBV DNA and subsequently linked to treatment if eligible per NVCHP guidelines.
- Vaccination against HBV was provided to all participants who were deemed susceptible to HBV (those testing negative for HBsAg and reported not previously vaccinated against HBV) according to the rapid vaccination regimen recommended by the World Health Organization.

The peer support staff of CoNE provided all participants education and counselling regarding prevention and treatment of HCV, HBV, and HIV.

#### Compliance management

All the participants were staying at the respective DTC during the entire course of DAA treatment. They were regularly counselled, one-to-one basis, about treatment process, need for adherence and cure by the peer support staff of CoNE and the DTC.

### Statistical analysis and sample size calculation

As this study did not have a testable hypothesis the sample size was derived from pragmatic considerations of ability to provide quality care to study participants in line with the funding availability for the study. Therefore, the study aimed to reach up to 600 PWID in DTC. To reach 600 participants for screening, 24 awareness camps were conducted in three months. Simple descriptive statistics were used to characterize participants and the number at each step of the care cascade. Sub-analyses were performed by age group. The data analysis was performed with excel 365.

#### Outcome measures

Primary outcome measure was proportion of HCV RNA detectable participants who could be initiated on antiviral treatment the same day. Secondary outcome measures were number of participants eligible for HBV vaccination, median time taken from HCV Ab screening test to start of HCV treatment, and proportion of those who were started on HCV treatment and could complete the treatment. Treatment success was defined as sustained virological response at 12 weeks, i.e., undetectable HCV RNA at 12 weeks after completing the treatment (SVR12). We measured SVR12 as per protocol analysis and intention to treat analysis.

## Results

All the 643 PWID who participated in the awareness camps in the 22 DTC were invited to participate in test and treat program, 540 (84%) of them agreed to participate, of those 503 met the eligibility criteria, and were included in study.

Of the 503 participants (males 100%; age median [IQR] 27 [23–32] years). 221 (43.9%), participants were only HCV Ab reactive, 9 were only HBsAg positive (1.8%), and 2 were only HIV reactive (0.4%). 21 participants (4.2%) were reactive on at least two tests (Table 1).

**Table 1:** Participants and type of infections

Total undergoing RDT testing		HCV Ab +		HBsAg +		HIV +		HCV Ab + & HBsAg +		HCV Ab + & HIV +		HBsAg + & HIV +		HCV Ab + , HBsAg + , HIV +	
Age	n	N	%*	n	%*	n	%*	n	%*	n	%*	n	%*	n	%*
18–19	30	15	50.0	0	0.0	0	0.0	2	6.7	0	0.0	1	3.3	0	0.0
20–24	148	74	50.0	4	2.7	0	0.0	6	4.1	0	0.0	0	0.0	0	0.0
25–29	159	83	52.2	2	1.3	0	0.0	3	1.9	1	0.6	0	0.0	0	0.0
30–34	69	20	29.0	1	1.4	0	0.0	2	2.9	0	0.0	0	0.0	1	1.4
35–39	40	16	40.0	0	0.0	0	0.0	1	2.5	0	0.0	0	0.0	0	0.0
40–44	33	7	21.2	1	3.0	0	0.0	1	3.0	1	3.0	0	0.0	1	3.0
45–49	16	5	31.3	1	6.3	2	12.5	0	0.0	0	0.0	0	0.0	0	0.0
50–54	6	1	16.7	0	0.0	0	0.0	0	0.0	1	16.7	0	0.0	0	0.0
55–60	2	0	0.0	0	0.0	0	0.0	0	0.0	0	0.0	0	0.0	0	0.0
Total	503	221	43.9	9	1.8	2	0.4	15	3.0	3	0.6	1	0.2	2	0.4

*% is per age grouping

Of the 8 participants with a reactive HIV RDT result, 5 were already on antiretroviral therapy and the remaining 3 were linked to the state HIV program, of those 2 initiated on ART and one did not consent to initiate on ART.

The 241 participants who had any combination of HCV Ab reactive results were evaluated further for HCV RNA and their clinical and laboratory characteristics and viremia was confirmed in 156 (64.7%).

Of those with detectable HCV RNA, 5 (3.2%) participants had cirrhosis based on APRI > 2.0. The median age [IQR] of those with cirrhosis was 34 [27–41.5] whereas the median age [IQR] of the 151 participants without cirrhosis was 25 [22–28].

Barring 3 participants, who were both HCV Ab reactive and HBsAg positive and had to wait for HBV DNA to be initiated on HCV treatment as per national guidelines, 153 of 156 viremic (99.4%) participants-initiated treatment on the same day with direct-acting antivirals. Two of the 3 participants who were both HCV Ab reactive and HBsAg positive were initiated on HCV treatment after their HBV DNA test and review by a physician, the 3rd participant was lost to follow up and was not initiated on either HCV or HBV treatment.

**Fig. 4:**
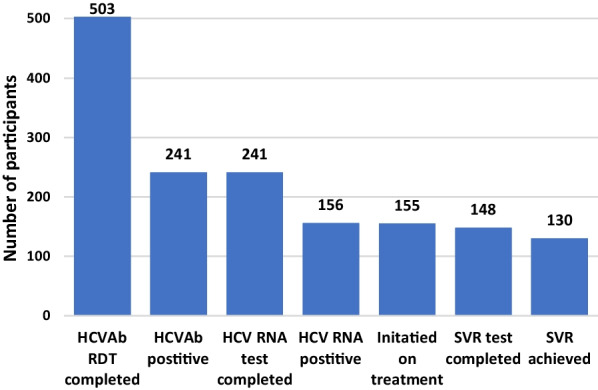
HCV care cascade

In between treatment completion and SVR 12 test, 7 participants were lost to follow-up including 2 participants who died due to unrelated causes after completion of treatment. Of the 148 tested for SVR12, samples of whom were collected through venipuncture by the peer PWID and sent to the same offsite laboratory for HCV RNA testing using Truenat® HCV, 130 had achieved SVR12. The treatment was successful in 87.8% and 83.9% in per-protocol and intention to treat analysis (Fig. 4). Among those participants who were treated for HCV and had cirrhosis, 4 of the 5 completed an SVR test and all 4 attained SVR. Of the 155 who were non-cirrhotic, 144 (92.9%) completed an SVR test and 126 (89.6%) of those achieved SVR12. The SVR 12 test results were collected by the peer PWID and delivered either at the home or at the DTC, if the participant was still undergoing drug use treatment. Of the 18 with failed SVR12, one was a person living with HIV. We were able to ascertain that nine had continued to use drugs after the treatment but this information was not available for the remaining nine participants. All participants with SVR 12 results were provided post treatment counselling, prevention of re-infection, liver care and possibility of re-treatment, if needed.

### Turn-around time analysis

All the 241 participants who were HCV Ab reactive were successfully tested (100%) for HCV RNA. The median time between HCV Ab rapid screening reactive result and HCV RNA testing was 38 min (IQR 30 min to 51 min). Median time between HCV Ab rapid screening reactive result and treatment initiation was 6 h 38 min (IQR 5 h 41 min- 8 h 23 min) (Table 2).

**Table 2:** Turn-around time between RDT testing and treatment initiation *RDT; rapid diagnostic test grouping

Time between	n	Median	Min	Max	IQR 1	IQR 3
HCV rapid screening test result & HCV RNA testing initiation	241	38 min	7 min	1 h 36 min	30 min	51 min
HCV rapid screening test result & treatment initiation	153	6 h 38 min	4 h 42 min	12 h 18 min	5 h 41 min	8 h 23 min

### HBV vaccination analysis

Of the 474 participants who were HBsAg RDT negative 31 (6.5%) had already been vaccinated for HBV meaning that 443 were eligible for vaccination, of those 7 (1.6%) did not receive any does of the HBV vaccine as 6 were discharged from the DTC and could not be reached and 1 has been on HBV treatment since 2019 despite testing HBsAg negative in this study. 436 (98.4% of those eligible for vaccination) received all 3 doses.

## Discussion

This study investigated a first-of-its-kind simplified model for Community-led comprehensive viral hepatitis care among vulnerable groups in India. Our findings demonstrate that the approach, which incorporates same day “test and treat” for HCV, is feasible and effective.

The simplified same day “test and treat” algorithm improved linkages from HCV diagnosis to treatment initiation, with all participants reactive for HCV Ab rapid screening linked for confirmatory HCV RNA testing, and 99.4% of those positive for HCV RNA linked to treatment on the same day. Prior to the intervention, around 50% of PWID and PLHIV in Manipur were lost between screening and confirmatory testing, and another 45% were lost in between diagnosis and treatment [10]. Cirrhosis was observed in 3.2% of the participants which had detectable HCV RNA. During the post-test counselling and course of treatment they were provided with information on the need for continued monitoring and management of their liver disease by the peer PWID.

Estimated burden of HCV among PWID in India is approximately ~ 440,000 [14]. A recent nation-wide survey, conducted to assess the burden of substance use disorder in the country, estimated the total number of PWID as 850,000; further, almost 50% and 27% of them reported syringe/needle reuse and sharing among themselves. A very small proportion of PWID in India seek access to preventive health care and are screened or vaccinated for viral hepatitis with only about 12% of PWID reported ever receiving any help or treatment. [12].

The need for repeated visits to health care facility between screening and confirmatory tests as well as initiation of treatment could result in patients not completing cascade of care as seen in one study in Delhi in which the loss to follow up from screening to HCV RNA confirmatory testing ranged from 6.3% in the arm which used reflex sample referral to 47.5% in the arm in which patients were referred from the site where screening was conducted to a separate location for RNA testing [15].

One community based, supervised HCV treatment experience in 3477 PWID in India, showed that only 65.5% could complete the treatment and 57% could be tested for SVR12. The SVR12 rates were 91.1% and 49.5% on per-protocol analysis and intention to treat analysis respectively [16]. However in another study of a one stop shop approach of HCV care among PWID 86.9% achieved SVR12 [17]. Our approach of integrated test and treat strategy, supervised therapy and supported by peer PWID showed better SVR12 rates at 87.8% and 83.9% in per-protocol and intention to treat analysis. This suggest that integration of screening test, confirmatory test, and initiation of treatment followed by regular supervision by peers could achieve a high success rate among high risk groups.

A same day “test and treat” model for HCV and HBV in Egypt also found the approach to be feasible and able to achieve high levels of linkage to care and treatment. In this study, key portable laboratory instruments (GeneXpert®, FibroScan® and abdominal ultrasound) were transported to the community sites for screening [18]. Another study looking at same day test and treat among PWIDs in rural Malaysia used a GeneXpert® that shifted between clinics, in that study 71.1% (108/152) participants started treatment on the same day of the HCV RNA testing [19]. In our study, we demonstrated the feasibility of a simpler same day “test and treat” approach for HCV which does not require for the HCV RNA testing platform to be located on site by leveraging sample referral and coordinated peer navigation. Our study used the near point-of-care molecular testing platform, Truenat®^.^ The Truenat® platform is made in India and can be run using minimal infrastructure, by minimally trained technicians and provides an end-to-end solution from sample collection to reporting of results, with results available in under an hour [20]. Consequently, the Truenat® platform provides a way to realize same-day “test and treat” models of hepatitis care, and offers an alternative to GeneXpert® that may be better suited for use in low- and middle-income countries.

Though harm reduction programs such as, opioid substitution treatment and needle syringe program, are in place, the risk and burden of HCV infection among PWID is still very high. Globally, 52.3% PWID have evidence of ongoing or past HCV infection [21]. There are obstacles, which operates at the level of participants, service provider, and health care system which prohibits HCV elimination in PWID. The prominent issues are their poor access and inadequate interest by health care provider; inadequate self-motivation, poor knowledge, and social challenges faced by the PWID to participate in health care programs; these problems are markedly aggravated with risks of poor adherence to testing and treatment protocol, and of repeated infections following treatment [22]. All around the world, treatment adherence and completion had known to be a major issue challenge for PWID living with HCV. Several approaches, centred at the level of participant, service provider, or heath care system have been applied to enhance the treatment adherence and improve the linkage with cascade of care [23].

## Conclusion

Same day “test and treat” approaches for HCV hold great potential to close gaps in hepatitis C diagnosis and treatment, especially for high risk groups who may face additional challenges when trying to access care and may assist in reaching the WHO 2030 targets on HCV elimination of 90% of persons living with HCV knowing their status and 80% of those treated among PWID. Creating non-stigmatizing, streamlined care and a supportive healthcare system and social environment are essential for marginalized groups like PWID to freely and openly access viral hepatitis services. The PWID peer support extended resulted in ensuring compliance to care and treatment cascade and completing all the three doses of HBV vaccination. The availability of near point-of-care platforms such as Truenat® HCV contributes to the potential of reaching viral hepatitis elimination targets. Our model can be replicated or adapted for other settings to increase equity in access to HCV and HBV prevention, screening, and treatment.

## Data Availability

De-identified participant data will be made available on appropriate request to the corresponding author.

## References

[1] Global progress report on HIV, viral hepatitis and sexually transmitted infections, 2021. Accountability for the global health sector strategies 2016–2021: actions for impact. Geneva: World Health Organization; 2021. Licence: CC BY-NC-SA 3.0 IGO.

[2] WHO guidelines on hepatitis B and C testing. Geneva: World Health Organization; 2017. Licence: CC BY-NC-SA 3.0 IGO.

[3] Premkumar M, Kumar CY (2021). Chronic Hepatitis B: challenges and successes in India. Clin Liver Dis.

[4] Dhiman RK (2014). Future of therapy for Hepatitis C in India: a matter of accessibility and affordability?. J Clin Exp Hepatol.

[5] Ministry of Health and Family Welfare Government of India, National Action Plan Combating Viral Hepatitis in India. 2019. https://www.who.int/docs/default-source/primary-health-care-conference/national-action-plan-lowress-reference-file.pdf?sfvrsn=6a00ecbf_2. Accessed 18 February 2022.

[6] Karam RS, Singh TD, Akoijam BS, Rajkumar N (2021). Viral hepatitis C infection among injecting drug users and their partners in Manipur. J Med Evid.

[7] Eicher AD, Crofts N, Benjamin S, Deutschmann P, Rodger AJ (2000). A certain fate: spread of HIV among young injecting drug users in Manipur. North-East India AIDS Care.

[8] Solomon SS, Mehta SH, Srikrishnan AK, Solomon S, McFall AM, Laeyendecker O (2015). Burden of hepatitis C virus disease and access to hepatitis C virus services in people who inject drugs in India: a cross-sectional study. Lancet Infect Dis.

[9] Kermode M, Longleng V, Singh BC (2007). My first time: initiation into injecting drug use in Manipur and Nagaland, north-east India. Harm Reduct J.

[10] Community Network for Empowerment (CoNE), Manipur. Internal hepatitis C cascade of care data from 2014 to May 2021.

[11] Fourati S, Feld JJ, Chevaliez S, Luhmann N (2018). Approaches for simplified HCV diagnostic algorithms. J Int AIDS Soc.

[12] Ambekar A, Agrawal A, Rao R, Mishra AK, Khandelwal SK, Chadda RK on behalf of the group of investigators for the National Survey on Extent and Pattern of Substance Use in India (2019). Magnitude of Substance Use in India. New Delhi: Ministry of Social Justice and Empowerment, Government of India.

[13] Kermode M, Nuken A, Medhi GK, Akoijam BS, Sharma HU, Mahanta J (2016). High burden of hepatitis C & HIV co-infection among people who inject drugs in Manipur. Northeast India Indian J Med Res.

[14] Goel A, Rewari BB, Sharma M, Konath NM, Aggarwal R (2022). Seroprevalence and burden of hepatitis C virus infection in WHO South-East Asia Region: a systematic review. J Gastroenterol Hepatol.

[15] Markby J, Gupta E, Soni D, Sarin S, Murya M, Katapur P (2022). Feasibility, effectiveness and cost of a decentralized HCV care model among the general population in Delhi. India Liver Int.

[16] Dhiman RK, Grover GS, Premkumar M, Roy A, Taneja S, Duseja A, MMPHCRF Investigators (2021). Outcomes of real-world integrated HCV microelimination for people who inject drugs: an expansion of the Punjab model. EClinicalMedicine.

[17] Markby J, Sarin S, Soni D, Maurya M, Babu ER, Tewati N et al. Retention in the HCV Care Cascade for People Living with HIV In Delhi And Manipur, India And Malaysia: The HEAD-Start Project. Reviews in Antiviral Therapy & Infectious Diseases 6, 2020. Asia-Pacific AIDS & Co-Infections Conference 2020 Abstract 118.

[18] Shiha G, Soliman R, Serwah A, Mikhail NNH, Asselah T, Easterbrook P (2020). A same day 'test and treat' model for chronic HCV and HBV infection: results from two community-based pilot studies in Egypt. J Viral Hepat.

[19] Hassan MRA, Chan HK, Nordin M, Yahya R, Sulaiman WRW, Merican SAA (2023). Assessing feasibility of a modified same-day test-and-treat model for hepatitis C among rural people who inject drugs. Harm Reduct J.

[20] WHO consolidated guidelines on tuberculosis. Module 3: diagnosis. Tests for tuberculosis infection. Geneva: World Health Organization; 2022. Licence: CC BY-NC-SA 3.0 IGO 36441853

[21] Grebely J, Larney S, Peacock A, Colledge S, Leung J, Hickman M (2019). Global, regional, and country-level estimates of hepatitis C infection among people who have recently injected drugs. Addiction.

[22] Schwarz T, Horváth I, Fenz L, Schmutterer I, Rosian-Schikuta I, Mårdh O (2022). Interventions to increase linkage to care and adherence to treatment for hepatitis C among people who inject drugs: a systematic review and practical considerations from an expert panel consultation. Int J Drug Policy.

[23] Cunningham EB, Wheeler A, Hajarizadeh B, French CE, Roche R, Marshall AD (2023). Interventions to enhance testing and linkage to treatment for hepatitis C infection for people who inject drugs: a systematic review and meta-analysis. Int J Drug Policy.

